# Efficacy of Superoxide Dismutase in the Treatment of Vitiligo: A Systematic Review

**DOI:** 10.7759/cureus.88335

**Published:** 2025-07-19

**Authors:** Virginia Salazar-Formoso, Martha Alejandra Morales-Sánchez

**Affiliations:** 1 Dermatology, Centro Dermatológico "Dr. Ladislao de la Pascua", IMSS para el Bienestar, Mexico City, MEX

**Keywords:** oxidative stress, repigmentation therapy, superoxide dismutase, treatment, vitiligo

## Abstract

Vitiligo is a chronic autoimmune skin condition characterized by the progressive loss of melanocytes, resulting in well-defined hypopigmented or achromic patches. Oxidative stress is believed to play a role in its pathogenesis, and superoxide dismutase (SOD), an antioxidant enzyme, has emerged as a potential therapeutic option. This systematic review aimed to evaluate the efficacy of oral or topical SOD, used either alone or in combination with other interventions, in the treatment of vitiligo. Following the Preferred Reporting Items for Systematic reviews and Meta-analyses (PRISMA) 2020 guidelines, a comprehensive search of PubMed (MEDLINE), Cochrane, ClinicalTrials.gov, EBSCO, LILACS, Scopus, and Tesis UNAM was conducted, and randomized clinical trials published up to April 14, 2025, were included. Eight randomized controlled trials (RCTs) were analyzed, including four with split-body and four with parallel-group designs. SOD was administered either alone or in combination with other therapies such as narrowband ultraviolet B (NB-UVB) phototherapy, excimer lamp, or calcineurin inhibitors. The primary outcome assessed across studies was the percentage of repigmentation. Six studies found no significant difference in repigmentation compared to controls, while two reported improved outcomes when SOD was combined with NB-UVB in responsive areas. One study showed a significantly greater improvement in the Vitiligo Extent Score (19.85% vs. 8.83%), and another reported excellent or good repigmentation in 46.7% of lesions with combination therapy, compared to none in the monotherapy group. All studies described a favorable safety profile. The risk of bias was low in four studies, some concerns were identified in four studies, and three studies were rated as high risk, primarily due to the lack of blinding of participants and outcome assessors, as well as unclear allocation concealment and randomization procedures. Although SOD appears to be well-tolerated and has demonstrated a favorable safety profile across all included studies, its efficacy remains uncertain. Only two studies reported modest improvements in repigmentation, both in combination with NB-UVB, making it difficult to isolate the contribution of SOD. While current evidence does not support the use of SOD as a standalone or first-line treatment, it may have potential as an adjunctive option. Further high-quality trials are needed to clarify its role in clinical practice, particularly in combination regimens.

## Introduction and background

Vitiligo is an acquired, autoimmune, chronic dermatosis of multifactorial origin, characterized by the selective and progressive loss of melanocytes (the cells responsible for producing skin pigment) in the epidermis and hair follicles, resulting in well-demarcated hypochromic or achromic patches [1]. It is the most common hypopigmentation disorder, affecting 0.1-2% of the global population. In most cases, it presents before the age of 20, although it can appear at any point throughout life [1-3].

The exact pathogenesis of vitiligo remains unknown, but increased oxidative stress and the accumulation of melanocytotoxic compounds have been proposed as possible mechanisms for the disappearance of functional melanocytes in the affected skin [2]. In patients with vitiligo, elevated levels of hydrogen peroxide (H₂O₂) have been detected in the epidermis. This oxidative overload can inactivate catalase, one of the key enzymes responsible for detoxifying H₂O₂, leading to a vicious cycle of oxidative stress [3]. Superoxide dismutase (SOD) plays a complementary role by converting superoxide radicals, highly reactive and damaging species, into H₂O₂ and O₂. While this reaction increases H₂O₂ transiently, the combined action of SOD and catalase is essential for maintaining redox balance under normal conditions. In vitiligo, the dysfunction of this antioxidant network supports the hypothesis of an imbalance between reactive oxygen species and protective enzymes [3].

Despite the available treatments, vitiligo remains a therapeutic challenge, partly due to its unpredictable course, variable response to therapies, and its significant psychosocial impact on patients’ quality of life [1]. Recent treatments aim to address the imbalance in the antioxidant system, which is why the use of the enzyme SOD, both orally and topically, has been proposed [4]. This enzyme acts by reducing oxidative stress and decreasing the activation of inflammatory mediators [5]. Due to the relatively recent recognition of oxidative stress as a key factor in vitiligo pathogenesis, particularly over the past two decades, the introduction of antioxidant-based therapies like SOD is still considered novel and under active investigation.

Due to the recent introduction of SOD-based therapies, either as monotherapy or in combination with other treatments, there is still limited evidence regarding their efficacy and superiority compared to established vitiligo treatments. Although several clinical trials have explored the use of oral or topical SOD in vitiligo, to our knowledge, no prior systematic review has synthesized the available evidence. Therefore, the aim of this study was to conduct an analysis of all randomized clinical trials evaluating the efficacy of SOD, administered either orally or topically, used as monotherapy or in combination with other therapies, in the repigmentation of lesions in patients with vitiligo, in order to provide more certainty and information for future therapeutic decision-making.

## Review

Methods

Study Design

A literature review was conducted in accordance with the Preferred Reporting Items for Systematic reviews and Meta-analyses (PRISMA) 2020 guidelines, with the objective of evaluating SOD for the treatment of vitiligo. The protocol was registered in PROSPERO under the registration number: CRD420251034529. The search was conducted by two independent reviewers, and a third reviewer assisted in case of discrepancies. The search was carried out using the following databases and search engines: PubMed (MEDLINE), Cochrane, Clinical Trials, EBSCO, LILACS, Scopus, and Tesis UNAM, using the following medical subject headings (MeSH) terms or keywords: “vitiligo,” “superoxide dismutase,” and “treatment.”

Inclusion Criteria

This review included randomized controlled trials (RCTs) conducted in humans clinically diagnosed with nonsegmental vitiligo, regardless of geographical location, published and conducted up to April 14, 2025, in any language.

Exclusion Criteria

Quasi-experimental studies, commentaries, letters to the editor, case reports, case series, conference abstracts, narrative reviews, and articles not available in full-text version were excluded.

The information obtained was analyzed according to the previously defined inclusion and exclusion criteria. The effects of SOD on the repigmentation of vitiligo lesions compared to various standard treatments were summarized in Table 1.

**Table 1:** Summary of randomized controlled trials evaluating the efficacy of superoxide dismutase in vitiligo treatment UVB: ultraviolet B

Author	Year	Title	Country	Duration (months)	Total population	Intervention	Comparator	Outcome measure	Result
Naini et al. [5]	2012	The effect of pseudocatalase/superoxide dismutase in treatment of vitiligo: a pilot study	Iran	6	23	Topical pseudocatalase/superoxide dismutase	Topical placebo	% of repigmentation	No superiority observed
Alshiyab et al. [6]	2022	Comparison of the efficacy of tacrolimus 0.1% ointment and tacrolimus 0.1% plus topical pseudocatalase/superoxide dismutase gel in children with limited vitiligo: a randomized controlled trial	Jordan	9	49	Topical tacrolimus 0.1% + topical pseudocatalase/superoxide dismutase	Topical tacrolimus 0.1%	% of repigmentation	No superiority observed
Fontas et al. [7]	2021	Oral gliadin-protected superoxide dismutase in addition to phototherapy for treating non-segmental vitiligo: A 24-week prospective randomized placebo-controlled study	France	6	50	Oral gliadin-protected superoxide dismutase + narrowband UVB	Oral placebo + narrowband UVB	% of repigmentation	With superiority
Sanclemente et al. [8]	2008	A double-blind, randomized trial of 0.05% betamethasone vs. topical catalase/dismutase superoxide in vitiligo	Colombia	10	25	Topical pseudocatalase/superoxide dismutase	Topical betamethasone 0.05%	% of repigmentation	No superiority observed
Soliman et al. [9]	2016	Comparative study between excimer light and topical antioxidant versus excimer light alone for treatment of vitiligo	Egypt	3	30	Topical superoxide dismutase + narrowband UVB	Narrowband UVB	% of repigmentation	With superiority
Yuksel et al. [10]	2009	Comparison of the efficacy of narrow band ultraviolet B and narrow band ultraviolet B plus topical catalase-superoxide dismutase treatment in vitiligo patients	Turkey	6	30	Topical pseudocatalase/superoxide dismutase + narrowband UVB	Narrowband UVB	% of repigmentation	No superiority observed
Paracha et al. [11]	2010	Comparison of treatment with tacrolimus 0.03% and superoxide dismutase and catalase in vitiligo	Pakistan	6	60	Topical pseudocatalase/superoxide dismutase	Topical tacrolimus 0.03%	% of repigmentation	No superiority observed
Rahsepar et al. [12]	2022	Evaluation of the additional effect of Vitix® gel on vitiligo lesions in patients treated with narrow-band ultraviolet-B phototherapy	Iran	4	30	Topical pseudocatalase/superoxide dismutase + narrowband UVB	Narrowband UVB	% of repigmentation	No superiority observed

A meta-analysis was not performed due to significant heterogeneity among the included studies. This heterogeneity was primarily related to the formulation and route of administration of SOD (oral or topical), as well as the lack of detailed information regarding the specific chemical composition of the formulations. In addition, the studies used different comparative interventions, including narrowband UVB (NB-UVB) phototherapy, calcineurin inhibitors, topical corticosteroids, and a placebo. Treatment durations also varied across studies. Despite these differences, all studies assessed clinical response using similar outcome measures based on repigmentation percentage.

Results

**Figure 1:**
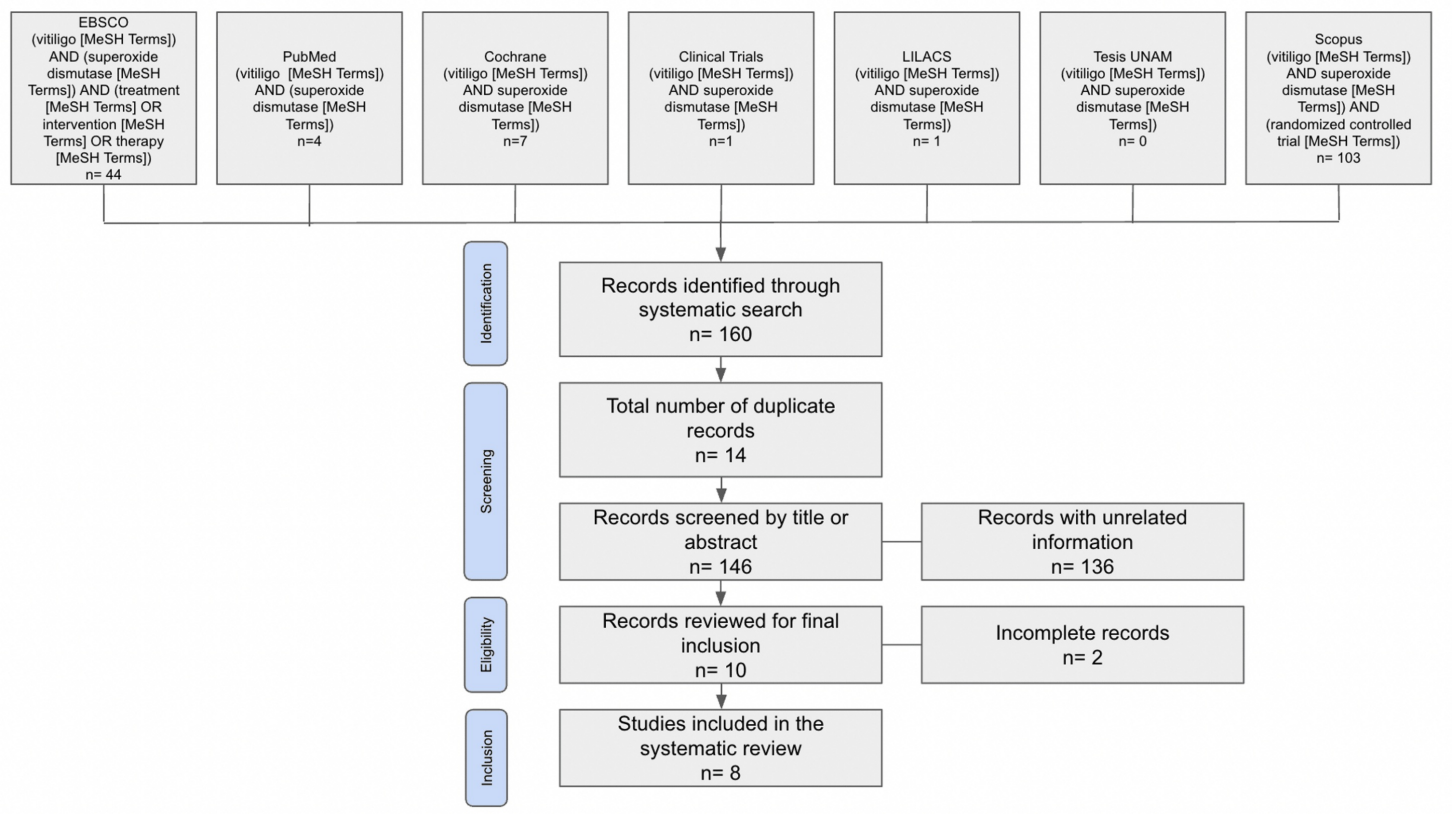
PRISMA 2020 flow diagram of the study selection process

Following the search across various databases, a total of 160 articles were initially identified. After removing 14 duplicates, 146 titles and abstracts were screened. Of these, 136 were excluded for not meeting the inclusion criteria. From the 10 remaining records, two were excluded due to the lack of access to the full text, leaving eight studies for final evaluation. Figure 1 illustrates the PRISMA 2020-compliant flow diagram of the selection process.

Eight studies published between 2008 and 2022 were included [5-12]. Two studies were conducted in Iran, and the remaining six were carried out in different countries: Jordan, France, Colombia, Egypt, Turkey, and Pakistan. Most of these studies were conducted in Asian countries (n = 4). All studies included in this systematic review were RCTs, of which four employed a conventional parallel-group design and four utilized a split-body intraindividual design, in which each patient received the experimental treatment on one side of the body and the placebo or standard therapy on the opposite side. The inclusion of split-body RCTs helped reduce interindividual variability and strengthen the evaluation of these therapies.

Most studies involved follow-up assessments at monthly intervals, with final evaluations ranging between 3 and 10 months. All studies assessed treatment efficacy based on the percentage of repigmentation of vitiligo lesions, measured using various methods such as subjective visual estimation by investigators, photographic comparison, standardized scoring systems like the Vitiligo Area Scoring Index (VASI), or patient self-assessment.

The studies evaluated the efficacy of SOD, administered either topically or orally, alone or in combination with other therapies such as phototherapy (NB-UVB) or calcineurin inhibitors (tacrolimus 0.1%). The intervention was compared to placebo, phototherapy, calcineurin inhibitors (tacrolimus 0.03% and 0.1%), and topical corticosteroids (betamethasone 0.05%).

The majority of studies (n = 6) did not demonstrate statistically significant superiority in repigmentation percentage with the use of SOD, whether alone or in combination, compared to control groups. This does not imply a complete lack of effect, but rather that the outcomes were not significantly better than those observed in the comparators. Four of these studies used a split-body design, which enabled more precise intraindividual comparisons by reducing inter-individual variability and controlling for confounding factors such as age, skin type, and disease duration. None of the studies reported relevant adverse effects associated with topical or oral SOD use, suggesting a favorable safety profile. In the studies where no additional benefit was observed, it was suggested that SOD might offer a comparable therapeutic effect, though without clinical superiority. This supports its use as a safe alternative, particularly for patients who do not tolerate other therapies.

Regarding the studies that demonstrated clinical superiority with SOD use, one was an RCT comparing NB-UVB phototherapy combined with placebo versus NB-UVB combined with oral SOD, formulated with a gliadin coating to prevent early gastrointestinal degradation. The results showed greater clinical efficacy in the combined treatment group, achieving 20% repigmentation after 24 weeks of oral SOD plus NB-UVB, compared to 9% repigmentation in the group receiving NB-UVB plus placebo. Additionally, most patients reported a significant improvement in quality of life.

The second study showing benefit used a split-body design with 30 patients, in which 2 to 4 lesions were randomly selected to receive topical SOD combined with excimer lamp or excimer lamp alone. The combined treatments resulted in a higher percentage of repigmentation, particularly in areas known to respond well to phototherapy (face, neck, trunk, and limbs). No statistically significant differences were observed in difficult-to-treat areas such as bony prominences and distal extremities, and better outcomes were noted in recently developed lesions. No significant adverse events were reported in either study.

Risk of bias was assessed using the Risk of Bias 2 (RoB 2) tool, which evaluates five key domains: random sequence generation, allocation concealment, blinding of participants and personnel, blinding of outcome assessors, and completeness of outcome data. Among the included studies, four were classified as having a low risk of bias, four showed “some concerns,” and three were judged to have a high risk of bias. A common limitation was the lack of blinding of participants and/or outcome assessors, which may have affected the objectivity of outcome measurements. Additionally, several studies did not clearly report their methods of allocation concealment or randomization, contributing to uncertainty in their internal validity. Given that repigmentation is a subjective outcome, this lack of blinding may introduce performance and detection bias, potentially leading to an overestimation of the interventions’ efficacy. Furthermore, incomplete reporting of randomization procedures raises concerns about selection bias due to possible baseline differences between groups. These methodological shortcomings reduce the certainty of the evidence and highlight the need for rigorously designed future trials with adequate blinding and transparent randomization methods (Table 2).

**Table 2:** RoB 2 evaluation of included RCTs on SOD-based therapies in vitiligo RoB: risk of bias; RCTs: randomized controlled trials; SOD: superoxide dismutase

Author	Randomization method	Allocation concealment	Blinding of participants & personnel	Blinding of outcome assessors	Incomplete outcome data	Overall risk of bias	Comments
Naini et al. [5]	Low risk	Low risk	Low risk	Low risk	Low risk	Low risk	Proper randomization, well-blinded, complete reporting
Alshiyab et al. [6]	Low risk	Some concerns	High risk	High risk	Low risk	Some concerns/high risk	Adequate randomization, no blinding of participants or assessors
Fontas et al. [7]	Low risk	Low risk	Low risk	Low risk	Low risk	Low risk	Double-blind, placebo-controlled, clear methodology
Sanclemente et al. [8]	Low risk	Low risk	Low risk	Low risk	Low risk	Low risk	Split-body study, well-blinded
Soliman et al. [9]	Some concerns	High risk	High risk	Low risk	Low risk	Some concerns/high risk	Randomization unclear, no participant blinding
Yuksel et al. [10]	Some concerns	Some concerns	High risk	Some concerns	Low risk	Some concerns	No description of randomization or blinding
Paracha et al. [11]	Low risk	High risk	High risk	High risk	Low risk	Some concerns/high risk	Randomization done, but no blinding; outcome reporting complete
Rahsepar et al. [12]	Low risk	Low risk	Low risk	Low risk	Low risk	Low risk	Split-body design; blinded outcome assessor; clear protocol and analysis

Discussion

This systematic review analyzed the evidence regarding the efficacy of SOD in the treatment of vitiligo compared to other standardized therapeutic measures. The information was extracted from eight RCTs, which lends greater scientific validity to the review [5-12]. Most of the included studies did not demonstrate clinical superiority with the use of topical or oral SOD when compared to placebo or conventional treatments [7,9]. However, the absence of demonstrated superiority does not imply a complete lack of therapeutic effect. In several cases, the outcomes observed in the SOD groups were comparable to those of standard treatments, and the lack of statistical significance may reflect limitations in study power, small sample sizes, or designs aimed at detecting superiority rather than noninferiority [5]. These findings suggest that SOD may still exert beneficial effects, even if not statistically superior, particularly when used in combination with established therapies. Regarding safety, no serious or treatment-limiting adverse effects were reported in either the intervention or control groups across the included studies. When adverse events were mentioned, they were minor, such as mild, self-resolving erythema [6]. However, only one study explicitly assessed adverse events using a standardized grading system (Common Terminology Criteria for Adverse Events (CTCAE)), and the rest did not systematically report safety outcomes [7]. This limits the ability to draw robust conclusions about the safety profile of SOD. While the absence of reported adverse effects may suggest good tolerability, this should be interpreted cautiously, as underreporting or lack of standardized assessment may mask less apparent risks. Therefore, we have avoided any claims of superior safety and instead characterize SOD as well-tolerated based on current, but limited, safety data.

The results align with the most recent hypothesis on the role of oxidative stress in the pathophysiology of vitiligo [2-4], suggesting that the persistence or emergence of new lesions is associated with elevated levels of reactive oxygen species and low levels of antioxidant enzymes [3,4]. In this context, SOD showed favorable outcomes in two of the analyzed trials [7,9]. One of the included studies demonstrated that oral administration of gliadin‑protected SOD, combined with narrowband UVB, achieved a mean repigmentation of 19.85% at 24 weeks, compared to 8.83% in the UVB-alone (placebo) arm. While the improvement within the SOD group was highly significant (p < 0.0001), the between-group difference (~11%) did not reach statistical significance (p = 0.089), possibly due to limited power and smaller-than-anticipated effect size [7]. The second study reported greater repigmentation in lesions treated with topical SOD and excimer lamp, with statistically significant results in areas known to respond well to phototherapy [9].

From a clinical standpoint, the findings of this review support the consideration of SOD as a safe adjunctive treatment in selected scenarios, even in the absence of demonstrated superiority [6]. Its favorable tolerability profile makes it particularly attractive for patients who are intolerant or contraindicated for conventional therapies, such as corticosteroids or calcineurin inhibitors, due to side effects, comorbidities, or long-term safety concerns [7]. Additionally, SOD might be considered as an add-on therapy in patients with localized lesions in cosmetically sensitive or phototherapy-responsive areas (e.g., face, neck, trunk), especially when rapid or enhanced repigmentation is desired [9]. Although not suitable as monotherapy or first-line treatment, SOD-based interventions may complement other modalities in an integrative approach, particularly when individualized patient factors limit standard options. Future clinical trials should explore these targeted indications to better define their role within personalized treatment algorithms.

One of the strengths of this review is the comprehensive search strategy implemented across multiple databases, focusing exclusively on RCTs. Notably, half of the included studies utilized a split-body design [5,8,9,12], which helps minimize interpatient variability and strengthens the reliability of the findings. While one study specifically targeted a pediatric population, allowing for preliminary extrapolation of results, this represents only a first step; further research dedicated to children with vitiligo is essential to draw more definitive conclusions and inform clinical practice in this subgroup [6]. Regarding limitations or areas for improvement, it is important to highlight the limited number of available studies [10], the lack of standardization in SOD formulations [11], and inconsistencies in the methods used to evaluate the percentage of repigmentation, which make comparisons between studies difficult [12]. One key issue is that the included studies used different formulations, some topical, others oral, with limited or no detail regarding the concentration of SOD, the vehicles used, or the presence of other active ingredients. For example, in topical preparations, the vehicle can significantly influence skin penetration and bioavailability, yet this was rarely specified. Similarly, the pharmacokinetics and systemic effects of oral formulations may differ substantially, making it difficult to attribute the observed effects solely to SOD. This heterogeneity in formulation and administration route complicates the interpretation of efficacy outcomes and limits reproducibility. In addition, the methods used to evaluate repigmentation varied across studies. Some relied on subjective visual estimation, while others used photographic comparisons, and only a few employed standardized scoring systems. These inconsistencies not only introduce potential observer bias but also hinder the ability to pool data or perform meta-analyses. Overall, these methodological limitations reduce the strength of the evidence and underscore the need for greater transparency and consistency in future trials. Additionally, the heterogeneity in patient populations, with inclusion of both recent and long-standing vitiligo lesions, adds complexity to the interpretation of results. Recent lesions tend to respond better to treatment due to a relatively preserved melanocyte reservoir and less chronic tissue damage, whereas long-standing lesions often show resistance because of melanocyte depletion and fibrosis. This variability underscores the importance of stratifying patients by lesion duration in future research [12].

The findings of this systematic review suggest that SOD may represent a safe therapeutic option as an adjunct in the management of vitiligo. However, the evidence did not show clinical superiority over other alternatives, and therefore, it should not be considered a stand-alone strategy or first-line treatment.

## Conclusions

This review evaluated the efficacy of topical or oral SOD in the treatment of vitiligo. The results suggest that patients may benefit from adjuvant therapy; however, only two studies showed superiority in the percentage of repigmentation compared to conventional treatments, and both involved combination therapy with NB-UVB. A noteworthy finding across all included studies was the consistently favorable safety profile of SOD, with minimal and generally mild adverse effects reported. This safety advantage represents a significant positive aspect of SOD as a therapeutic option for vitiligo, particularly given the chronic nature of the disease and the need for long-term treatment. Despite the valuable information provided by these studies, several were identified as having limitations related to participant blinding and proper randomization, which reduces the level of confidence in their results. Future RCTs with robust methodological design, larger sample sizes, and standardized interventions and outcome assessments are needed to more accurately determine the efficacy and safety of SOD in vitiligo treatment. Specifically, there is a need to standardize SOD formulations, include placebo-controlled comparisons, implement rigorous blinding of both treatment procedures and outcome evaluations, and provide explicit methodological descriptions for repigmentation measurement. Additionally, head-to-head trials comparing different SOD formulations or delivery methods, studies focusing on specific vitiligo subtypes or lesion characteristics, and cost-effectiveness analyses would offer valuable insights to optimize therapeutic strategies and guide clinical decision-making.
